# P05.13. Development of the phlegm syndrome questionnaire: a new instruction to assess traditional Chinese medicine syndrome for angina

**DOI:** 10.1186/1472-6882-12-S1-P373

**Published:** 2012-06-12

**Authors:** Y Guanlin, Z Huiyong, Z Zhe, Y Li, Y Changhe, D Ying, Q Wencheng, L Maoxin, W Xiayun, D Rui, C Zhihui

**Affiliations:** 1 Liaoning University of Traditional Chinese Medicine, Shenyang, China; 2 Affiliated Hospital of Liaoning University of TCM, Shenyang, China

## Purpose

The concept of ”syndrome” is not only used for diagnosis but also as an outcome to assess successful treatment in Traditional Chinese medicine (TCM). Integrated medicine assessment mode is based on the combination of disease outcome and syndrome. Seattle Angina Questionnaire (SAQ) is a kind of disease outcome, but there is no syndrome questionnaire available. The current syndrome outcome is based on the “Guidelines for clinical research on Chinese new herbal medicines”, which comes from the textbooks, and its psychometric properties have not been evaluated. This study is to develop a questionnaire to assess the phlegm syndrome for angina.

## Methods

Firstly, a nominal group composed of 7 TCM experts was organized to supervise the study. Secondly, phlegm symptoms were extracted from literature review (papers, textbooks and guidelines), and from in-depth interviews of 12 patients with angina and phlegm syndrome. Thirdly, items were generated from the phlegm symptoms with the following conditions: (1) not an angina symptom, e.g. chest pain; (2) not a diagnostic symptom, e.g. liking heavy greasy foods; (3) not unchangeable, e.g. obesity. (4) not about pulse and tongue. Each of the items was designated to a colloquial question through cognitive interviews with 3 patients and 3 healthy people. Finally, a draft questionnaire was evaluated in a pilot study of 20 paitents.

## Results

Twenty-one phlegm symptoms were extracted from literature review and patient interviews. Eight items were generated through discussions of the nominal group. According to the cognitive interviews, each item was designed into a single question, but one of the items was separated into two questions due to a double meaning. In the pilot test, two questions were modified with feedback from 11 patients. Finally, the draft questionnaire was developed now containing a total of 9 questions.

## Conclusion

The questionnaire can capture the phlegm syndrome for angina, and may be used as an outcome to assess TCM after the psychometric properties analysis.

